# The Effect of Intensity, Frequency, Duration and Volume of Physical Activity in Children and Adolescents on Skeletal Muscle Fitness: A Systematic Review and Meta-Analysis of Randomized Controlled Trials

**DOI:** 10.3390/ijerph18189640

**Published:** 2021-09-13

**Authors:** Chunchun Wu, Yongjin Xu, Zhaojing Chen, Yinhang Cao, Kehong Yu, Cong Huang

**Affiliations:** 1 Department of Sports and Exercise Science, College of Education, Zhejiang University, Hangzhou 310000, China; 107372@zju.edu.cn (C.W.); 22003045@zju.edu.cn (Y.X.); yukh@zju.edu.cn (K.Y.); 2 Department of Kinesiology, California State University San Bernardino, San Bernardino, CA 92407, USA; Zhaojing.Chen@csusb.edu; 3 School of Physical Education and Sport Training, Shanghai University of Sport, Shanghai 200438, China; caoyinhang@sus.edu.cn; 4 Department of Medicine and Science in Sports and Exercise, Tohoku University Graduate School of Medicine, Sendai 980-8575, Japan

**Keywords:** adolescents, children, meta-analysis, muscle fitness, physical activity

## Abstract

Physical activity could improve the muscle fitness of youth, but the systematic analysis of physical activity elements and muscle fitness was limited. This systematic review and meta-analysis aim to explore the influence of physical activity elements on muscle fitness in children and adolescents. We analyzed literature in Embase, EBSCO, Web of Science, and PubMed databases from January 2000 to September 2020. Only randomized controlled studies with an active control group, which examined at least 1 muscle fitness evaluation index in individuals aged 5–18 years were included. Articles were evaluated using the Jaded scale. Weighted-mean standardized mean differences (SMDs) were calculated using random-effects models. Twenty-one studies and 2267 subjects were included. Physical activity had moderate effects on improving muscle fitness (SMD: 0.58–0.96, *p* < 0.05). Physical activity element subgroup analysis showed that high-intensity (SMD 0.68–0.99, *p* < 0.05) physical activity <3 times/week (SMD 0.68–0.99, *p* < 0.05), and <60 min/session (SMD 0.66–0.76, *p* < 0.01) effectively improved muscle fitness. Resistance training of ≥3 sets/session (SMD 0.93–2.90, *p* < 0.01) and <10 repetitions/set (SMD 0.93–1.29, *p* < 0.05) significantly improved muscle fitness. Low-frequency, high-intensity, and short-duration physical activity more effectively improves muscle fitness in children and adolescents. The major limitation of this meta-analysis was the low quality of included studies. The study was registered in PROSPERO with the registration number CRD42020206963 and was funded mainly by the Ministry of Education of Humanities and Social Science project, China.

## 1. Introduction

Muscle fitness is an important embodiment of the health of children and adolescents [1] and is an independent factor in the prevention of chronic diseases [2]. Children and adolescents with higher levels of muscle fitness have a more favorable cardiovascular profile in later life [3,4]. In addition, low muscle fitness is associated with weak skeletal health [5], poor metabolism [6], and even inflammation [7] in children and adolescents, and is closely related to high risk of mortality in adulthood [8]. However, the lack of muscle fitness of children and adolescents is currently a global problem. In China, a nationwide study that tracked 1.5 million people showed that the muscle strength of children and adolescents has been declining for nearly 30 years [9]. Similarly, in developed countries, muscle explosives (vertical and long jump performance) in children and adolescents have steadily declined since the mid-1980s [10]. Swedish and Russian teenagers have shown a decline in muscle strength [11], while Canadian and Spanish children have shown a decline in grip strength [12] and standing long jump [13].

Physical activity is considered an effective adjustable factor for changing muscle fitness [14]. In children and adolescents, increased physical activity is an important way to improve muscle fitness. Nichols et al. found that 15 months of resistance training significantly increased leg strength in girls aged 14–17 years [15]. In another study, a 6-week suspension-training movement program increased the upper body muscle endurance in children [16]. Although these intervention studies have demonstrated the benefits of physical activity in improving muscle fitness, the effect of a single physical activity element has not been identified.

According to the recommendations of the American College of Sports Medicine (ACSM), physical activity should be developed based on the following elements: frequency, intensity, duration, type, and volume [17]. The current analysis of these elements is mainly focused on meta-analysis suggesting the dose–response relationship. For example, Urs Granacher and colleagues investigated the effects of balance training on balance performance in youth [18], young adult [19] and older adult [20]. The results suggested the best training period, frequency and volume for balance training in different populations. However, the effect of balance training on muscle fitness is small, so it is difficult to generalize the results to the relationship between different types of physical activity and muscle fitness. In addition, the characteristics of muscle fitness of different ages are different [21], so the results of adults may not be applicable to children and adolescents. Melanie Lesinski et al. [22] conducted a study on relationships between resistance training and physical performance in youth athletes, focusing on the most suitable training period, intensity, frequency, and volume of resistance exercise to improve young athletes’ performance. Specific resistance training is necessary for athletes with higher muscle fitness requirements, while for most teenagers, benefits can be gained by engaging in sufficient physical activity [23]. Thus, the optimal dose of physical activity to improve the muscle fitness may be different in youth. However, the current systematic research on physical activity elements and the muscle fitness of children and adolescents is scarce. Therefore, this study explored the influence of physical activity and numerous physical activity elements on children and adolescents’ muscle fitness, using muscle strength, endurance, and explosive power as the outcome indexes.

## 2. Materials and Methods

This meta-analysis was conducted following the recommendations of the Preferred Reporting Items for Systematic Reviews and Meta-Analyses (PRISMA) [24] and has been registered in the International System Review Prospective Register (PROSPERO) (CRD42020206963).

### 2.1. Literature Search

We performed a systematic literature search in PubMed, Web of Science, EBSCO, and Embase from January 2000 to September 2020. The following Boolean search syntax was used: ((exercise* OR activiti* OR train* OR sport*) AND (musc* fitness OR musc* strength OR musc* endurance OR musc* power OR musc* performance OR musc* function) AND (child* OR kid* OR adolescen* OR teen* OR youth OR puberty) AND (‘randomized controlled trial’ OR RCT)). In addition, the following filters were activated: text availability: full text; species: humans; ages: 5–18 years; languages: English. The search strategy used for the PubMed database was a combination of the MeSH database and Boolean search syntax, while the search syntax was adapted appropriately for searching the Web of Science.

### 2.2. Selection Criteria

Studies were considered eligible for inclusion if they provided relevant information on PICOS (participants, interventions, comparators, outcomes, and study design) and met the following inclusion criteria: (1) participants: healthy children and adolescents aged 5–18 years; (2) intervention: all types of physical activity intervention; (3) comparator: active or passive control groups (compared with the experimental group, the control group had a reduced training protocol or no training at all.); (4) outcome: at least one evaluation index of muscle fitness (i.e., muscle strength, muscle endurance, and muscle power); (5) study design: randomized controlled trials with pre-and post-measures. Studies were excluded when: (1) the trials contained adults; (2) interventions targeted specific groups of children, such as those with obesity or mental illness; (3) the physical activity involved using smart devices, such as mobile phones; (4) the study did not report enough data for effect size calculations. Based on the above criteria, two reviewers (C.W. and Y.X.) screened potentially relevant articles independently by analyzing the titles, abstracts, and full texts of the respective articles, to assess their eligibility. For the studies that were finally included, two reviewers (C.W. and Y.X.) collected data from reports independently to determine the data that could ultimately be used for meta-analysis.

### 2.3. Coding of Studies

All included studies were coded for certain variables: number of subjects, sex of subjects, age of subjects, participants, and training parameters (i.e., training time, training frequency, training intensity, and training volume). In particular, the included studies were divided into two categories according to their training protocol, namely resistance training and non-resistance training, when calculating the training volume. The training volume of resistance training was coded by the number of sets per exercise, the number of repetitions per set, and rest between sets, while the training volume of other types of training was expressed as metabolic equivalent of energy (MET) multiplied by the time and frequency per week (METs-min/week). The energy expenditure of each physical activity was coded according to the 2011 Compendium of Physical Activities [25] (Table S1). Furthermore, if a study reported progressive training, the mean training time, frequency, and intensity were computed [22].

To analyze the relationship between physical and muscle fitness, the indicators that reflect muscle fitness were mainly divided into three categories: muscle strength, muscle endurance, and muscle power. Only one representative outcome variable was included in the analysis when a study reported multiple outcomes of similar categories.

### 2.4. Assessment of Methodological Quality and Statistical Analyses

The methodological quality and risk of bias in all eligible studies were assessed using the Jadad Scale [26] in 3 aspects: randomization, double-blinding, and descriptions of dropouts and/or withdrawals (Y.X.). Each aspect was assigned a score of 0–2 points. A score of ≥ 3 was the cut-off score for studies with a low risk of bias. In addition, funnel plot was used to assign publication bias.

To assess the effectiveness of physical activity on muscle fitness and to establish relationships between physical activity elements and muscle fitness in children and adolescents, a random-effects model was used to weight the included studies according to the size of the standard deviation, and the weighted-mean standardized mean difference (SMD) was calculated. Quantitative data from the included studies were synthesized for meta-analysis using Review Manager V.5.3.5 (Copenhagen: The Nordic Cochrane Center, The Cochrane Collaboration, 2014). Results are presented as *p*-value, SMD, and 95% confidence interval (95% CI). At least two studies were required to calculate the effect of physical activity on muscle fitness, and to improve readability, the positive effect on muscle fitness results was expressed as a positive SMD. In addition, effect size values of SMD < 0.20 indicated trivial, 0.20 ≤ SMD < 0.50 indicated small, 0.50 ≤ SMD < 0.80 indicated medium, and SMD ≥ 0.80 indicated large effects [27].

According to the Cochrane Collaboration’s recommendation, the I^2^ statistic was used to evaluate heterogeneity among the included studies. An I^2^ of < 25% indicated low heterogeneity, 25% ≤ I^2^ ≤ 50% showed moderate heterogeneity, and I^2^ > 50% indicated high inconsistency [28]. If high inconsistency was found among studies, the reliability of the results was evaluated by sensitivity analysis. Funnel plot was used to evaluate publication bias.

## 3. Results

### 3.1. Study Characteristics

**Figure 1:**
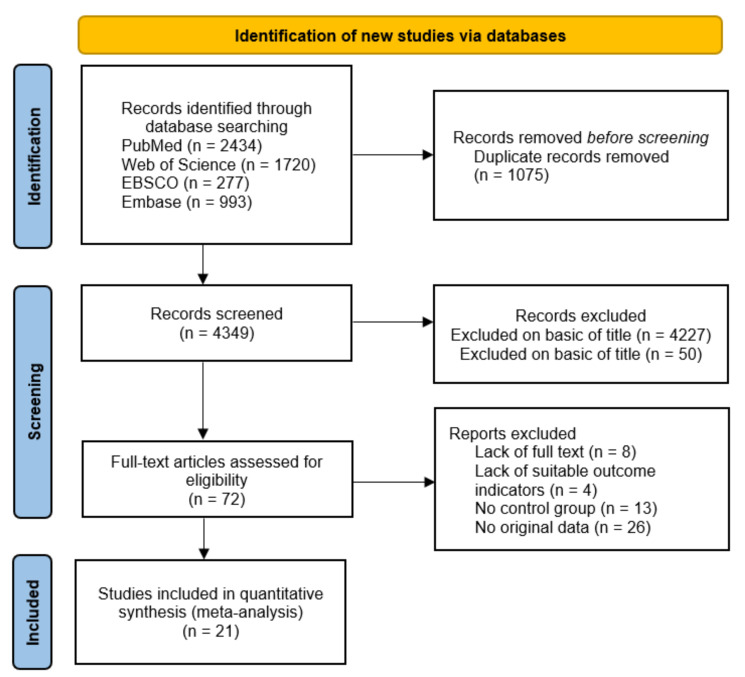
Flowchart illustrating the different phases of the search and study selection.

A total of 5424 potentially relevant studies were identified in the electronic databases PubMed, Web of Science, EBSCO, and Embase (Figure 1). After removing duplicates, screened through titles and abstracts, and excluding ineligible articles, 21 articles remained for quantitative analyses.

Table 1 shows the characteristics of all included literature. In the 21 articles overall, there were 27 intervention groups and 2267 subjects aged 5–18 years. Among these studies, interventions lasted between 4 and 60 weeks, with the training duration ranging from 4 to 60 weeks, training frequency ranging from 1 to 5 times per week, and the duration of a single intervention ranging from 3 to 60 min per session.

### 3.2. Methodological Quality and Bias Assessment

In general, the methodological quality of the included studies was classified as weak (Table 2). Of all the included studies, only one article mentioned double-blinding, and one article mentioned randomization and described specific methods, while the remaining articles did not implement blinding or did not describe specific randomization methods.

In the subgroup analysis, it was found that there was high heterogeneity among studies (I^2^ > 50%). However, due to the small number of studies included in the subgroup analysis, the source of the heterogeneity cannot be clarified, so the random effect model was used for analysis. In addition, sensitivity analysis was conducted by excluding the literature one by one, and the results showed that there was little difference from those without exclusion, suggesting low sensitivity, and the results were robust and credible. The funnel chart showed that a large number of studies were concentrated at the top, and a small number of studies were scattered, indicating that the publication bias is small (Figure S1).

### 3.3. Effects of Physical Activity

**Figure 2:**
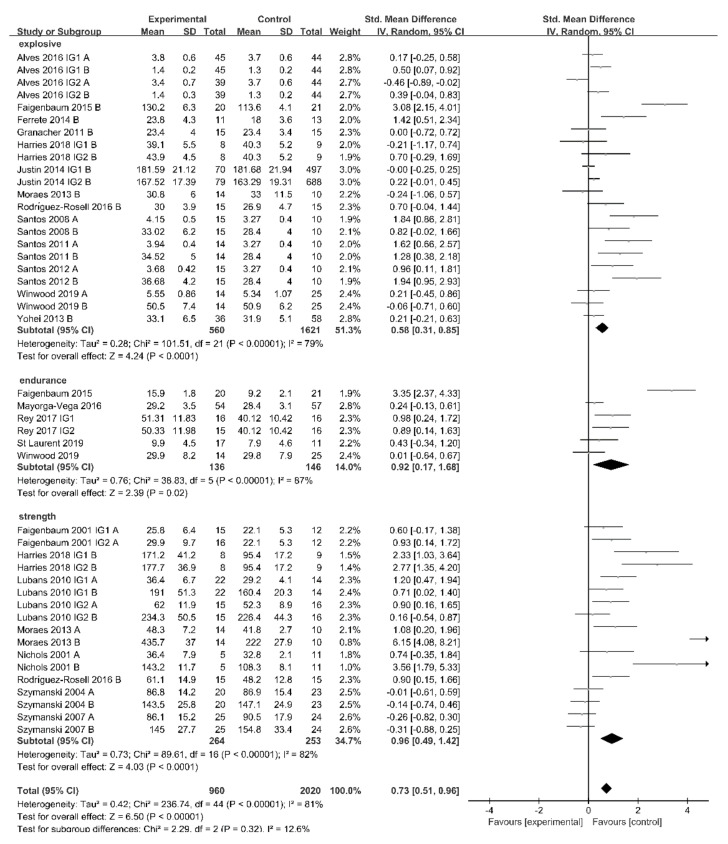
Effects of physical activity on muscle fitness. IG1: intervention group 1; IG2: intervention group 2; A: upper limb; B: lower limb.

Figure 2 illustrates the effects of physical activity on muscle fitness. The analysis revealed that physical activity plays an important role in enhancing muscle explosive power (SMD = 0.58 (0.31,0.85), I^2^ = 79%, χ^2^ = 101.51, df = 21, *p* < 0.0001), endurance (SMD = 0.92 (0.17,1.68), I^2^ = 87%, χ^2^ = 38.83, df = 5, *p* = 0.02), and strength (SMD = 0.96 (0.49, 1.42), I^2^ = 82%, χ^2^ = 81.69, df = 16, *p* < 0.0001).

**Figure 3:**
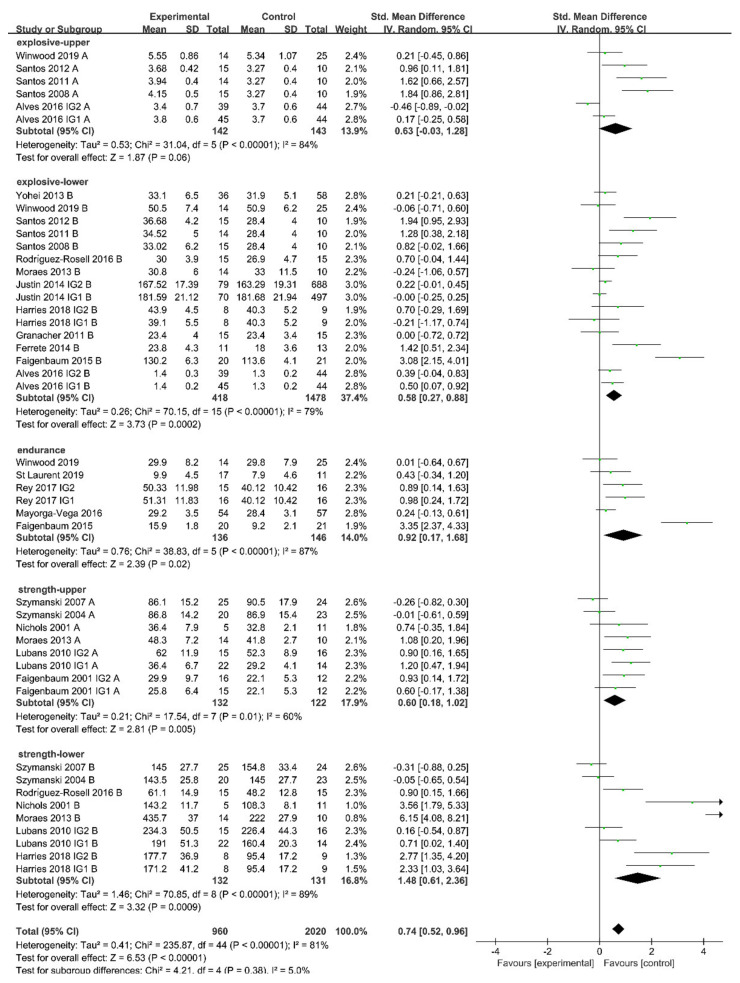
Effect of physical activity on the muscle strength and explosive of the upper and lower limb. IG1: intervention group 1; IG2: intervention group 2; A: upper limb; B: lower limb.

Furthermore, Figure 3 shows the effect of physical activity on the muscle strength and explosiveness of the upper and lower limb. The results show that physical activity can improve upper limb (SMD = 0.60 [0.18, 1.02], I^2^ = 60%, χ^2^ = 17.54, df = 7, *p* = 0.005) and lower limb (SMD = 1.48 (0.61, 2.36), I^2^ = 89%, χ^2^ = 70.85, df = 8, *p* = 0.0009) muscle strength, and lower limb explosive (SMD = 0.58 [0.27, 0.88], I^2^ = 79%, χ^2^ = 70.15, df = 15, *p* = 0.0002). The improvement in upper limb muscle explosive showed a tendency for statistical significance (SMD = 0.63 [−0.03, 1.28], I^2^ = 84%, χ^2^ = 31.04, df = 5, *p* = 0.06).

### 3.4. Effects of Physical Activity Elements

After clarifying the influence of physical activity on muscle fitness, we further analyzed the influence of various physical activity elements (i.e., training frequency, training intensity, training time, and training volume) through subgroup analysis.

#### 3.4.1. Training Frequency

**Figure 4:**
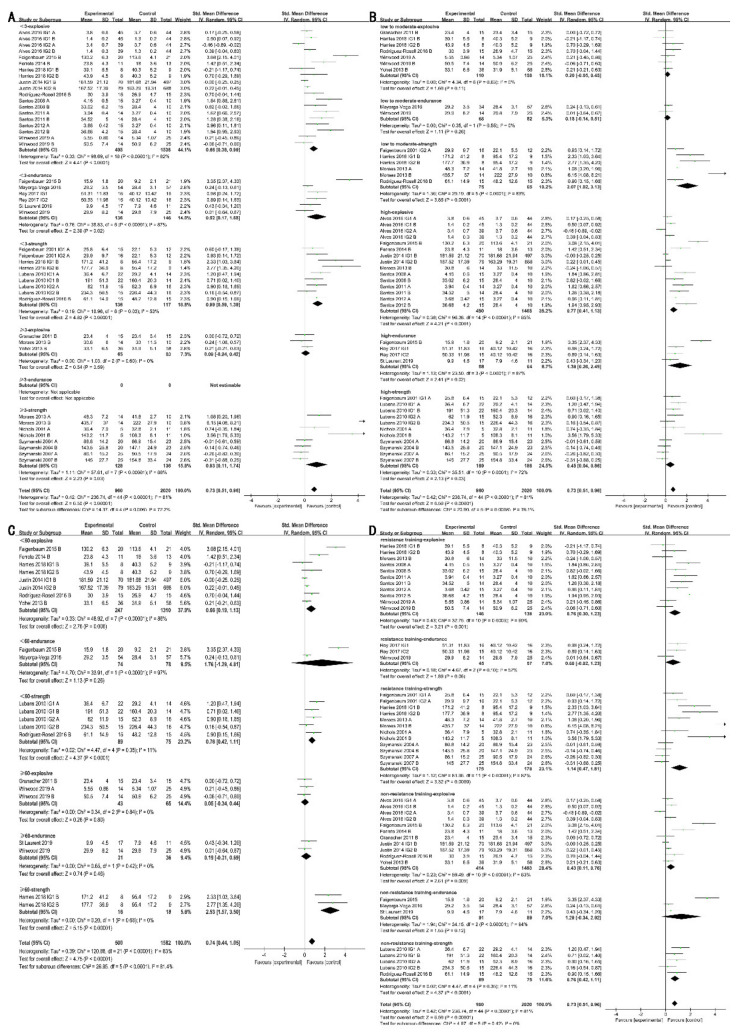
Effects of physical activity elements on muscle fitness. (**A**): Effects of training frequency on muscle fitness; (**B**): Effects of training intensity on muscle fitness; (**C**): Effects of training time on muscle fitness; (**D**): Effects of training type on muscle fitness; A: upper limb; B: lower limb.

There was a significant difference in the effects of physical activity on muscle strength regardless of training frequency (frequency < 3 time/week: SMD = 0.99 [0.59, 1.39], I^2^ = 53%, χ^2^ = 16.96, df = 8, *p* < 0.0001; frequency ≥ 3 time/week: SMD = 0.93 [0.11, 1.74], I^2^ = 88%, χ^2^ = 57.81, df = 7, *p* = 0.03; Figure 4A). Subgroup analysis indicated that low frequency (< 3 time/week) resulted in more pronounced improvements in muscle explosive power (SMD = 0.68 [0.38, 0.99]; I^2^ = 82%; χ^2^ = 98.69; df = 18; *p* < 0.0001) and endurance (SMD = 0.92 [0.17, 1.68]; I^2^ = 87%; χ^2^ = 38.83; df = 5; *p* = 0.02). However, the benefits of physical activity on muscle explosive power and endurance were not significant when the frequency was higher.

#### 3.4.2. Training Intensity

Physical activity only improved muscle strength significantly (SMD = 2.07 [1.02, 3.13]; I² = 83%; χ^2^ = 29.19; df = 5; *p* = 0.001) when performed at low-to-moderate intensity, while the improvement in explosive power and muscle endurance was not significant (Figure 4B). High-intensity physical activity resulted in pronounced improvements in muscle explosive power (SMD = 0.68 [0.38, 0.99]; I^2^ = 82%; χ^2^ = 98.69; df = 18; *p* < 0.0001), endurance (SMD = 0.92 [0.17, 1.68]; I^2^ = 87%; χ^2^ = 38.83; df = 5; *p* = 0.02), and strength (SMD = 0.99 [0.59, 1.39]; I^2^ = 53%; χ^2^ = 16.96; df = 8; *p* < 0.0001).

#### 3.4.3. Training Duration

Physical activity lasting less than 60 min appeared to be beneficial in improving muscle explosive power (SMD = 0.66 [0.19, 1.13]; I^2^ = 86%; χ^2^ = 48.92; df = 7; *p* = 0.006) and strength (SMD = 0.76 [0.42, 1.11]; I^2^ = 11%; χ^2^ = 4.47; df = 4; *p* < 0.0001; Figure 4C), while physical activity lasting more than 60 min only showed a significant enhancement of muscle strength (SMD = 2.53 [1.57,3.50]; I^2^ = 0%; χ^2^ = 0.20; df = 1; *p* < 0.0001).

#### 3.4.4. Training Type

Figure 4D illustrates that neither resistance training nor non-resistance training had a significant effect on muscle endurance, while both training types were beneficial in improving muscle strength (resistance training: SMD = 1.14 [0.47, 1.81]; I^2^ = 87%; χ^2^ = 81.96; df = 11; *p* = 0.0009; non-resistance training: SMD = 0.76 [0.42, 1.11]; I^2^ = 11%; χ^2^ = 4.47; df = 4; *p* < 0.0001) and explosive power (resistance training: SMD = 0.76 [0.30, 1.23]; I^2^ = 69%; χ^2^ = 32.75; df = 10; *p* = 0.001; non-resistance training: SMD = 0.43 [0.11, 0.76]; I^2^ = 83%; χ^2^ = 59.49; df = 10; *p* = 0.009).

#### 3.4.5. Training Volume

**Figure 5:**
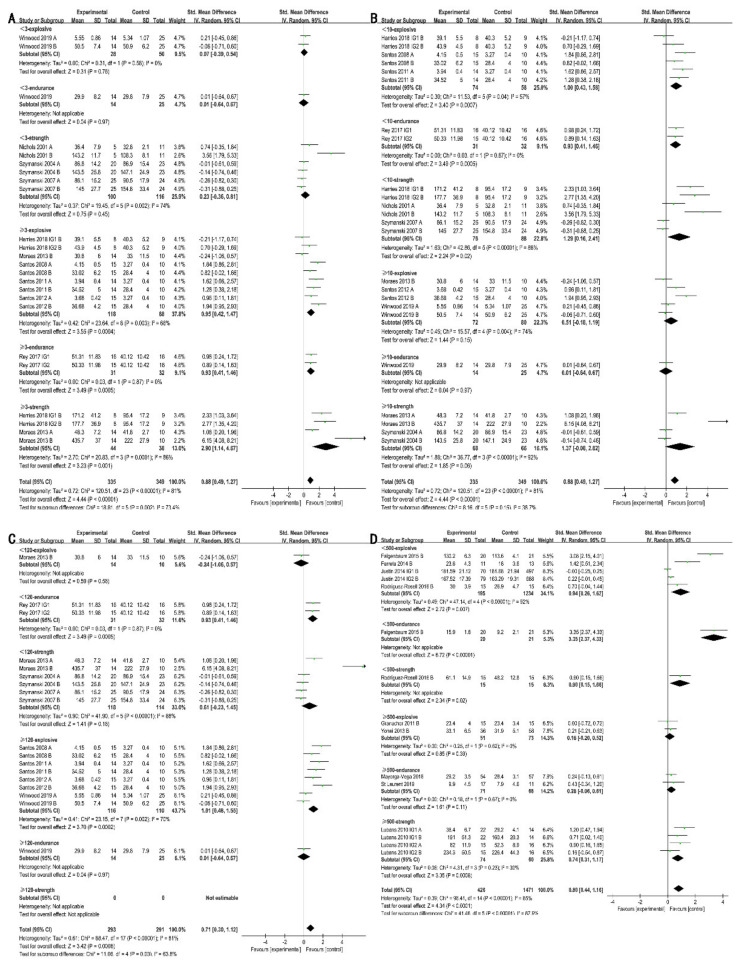
Effects of training volume on muscle fitness. (**A**): Effects of sets per session on muscle fitness; (**B**): Effects of repetitions per set on muscle fitness; (**C**): Effects of rests between sets on muscle fitness; (**D**): Effects of energy expenditure on muscle fitness; A: upper limb; B: lower limb.

Training volume was evaluated by sets per session, repetitions per set, and rest between sets for resistance training, while energy expenditure was utilized to estimate for non-resistance training. For sets per session, muscle explosive power (SMD = 0.95 [0.42, 1.47]; I^2^ = 66%; χ^2^ = 23.64; df = 8; *p* = 0.0004), endurance (SMD = 0.93 (0.41, 1.46); I^2^ = 0%; χ^2^ = 0.03; df = 8; *p* = 0.0004) and strength (SMD = 2.90 [1.14, 4.67]; I^2^ = 86%; χ^2^ = 20.83; df = 3; *p* = 0.001) improved significantly only with ≥ 3 sets/session (Figure 5A). For repetitions per set, the muscle explosive power (SMD = 1.00 [0.43, 1.58; I^2^ = 57%; χ^2^ = 11.53; df = 5; *p* = 0.0007), endurance (SMD = 0.93 [0.41, 1.46]; I^2^ = 0%; χ^2^ = 0.03; df = 1; *p* = 0.0005), and strength (SMD = 1.29 [0.16, 2.41]; I^2^ = 88%; χ^2^ = 42.86; df = 5; *p* = 0.02) improved significantly only with < 10 repetitions/set (Figure 5B). For rest between sets, improvement was only reflected in muscle endurance (SMD = 0.93 [0.41, 1.46]; I^2^ = 0%; χ^2^ = 0.03; df = 1; *p* = 0.0005) with rests shorter than 120 s; otherwise, it was only reflected in explosive power (SMD = 1.01 [0.48, 1.55]; I^2^ = 70%; χ^2^ = 23.15; df = 7; *p*=0.0002; Figure 5C). In addition, physical activity with energy expenditure < 500 METs-min/week resulted in improvement of muscle explosive power (SMD = 0.77 [0.23, 1.32]; I^2^ = 89%; χ^2^ = 47.22; df = 5; *p* = 0.005), and when weekly energy expenditure ≥ 500 METs, only muscle strength improved (SMD = 0.74 [0.31, 1.17]; I^2^ = 30%; χ^2^ = 4.31; df = 3; *p* = 0.0008; Figure 5D).

## 4. Discussion

This meta-analysis examined the impact of physical activity on the muscle fitness of children and adolescents and explored the effects of specific physical activity elements. In this study, we found that physical activity has a positive impact on the muscle fitness of children and adolescents, with a moderate impact on muscle explosive power, and a strong effect on muscle endurance and muscle strength. Moreover, we found that the more effective way to improve the muscle fitness of children and adolescents is by training less than three times per week, at high intensity, and for less than 60 min per session. Furthermore, if resistance training is used, a pattern of more than three sets and fewer than 10 repetitions per set is more effective. In addition, since the training program executed in the included literature was usually designed to be more than five repetitions, the number of repetitions per set can be more accurately targeted at 5–10.

### 4.1. Effects of Physical Activity on Muscle Fitness

In general, we found that physical activity can be used as an effective method to improve the muscle fitness of children and adolescents, which is consistent with the results of some previous studies [48]. In addition, our research showed that physical activity has a smaller effect on muscle explosive than muscle endurance and strength, which may be due to the influence of additional factors on explosive power training and testing. First, the magnitude of the force applied to this load affects the speed and power directly for the same absolute load [49]. Moreover, the increase in explosive power is also affected by training load, especially the fatigue level and speed loss in a set under the same relative intensity [50]. Second, the test of explosive power mainly uses medicine ball throwing or various jumping tests (i.e., countermovement jump, squat jump, and depth jump) [51], which places high demands on movement skills [51]. Therefore, performing such tests in children and adolescents may underestimate the actual muscle explosive power.

### 4.2. Effects of Physical Activity Elements on Muscle Fitness

#### 4.2.1. Training Frequency

Low-frequency physical activity seems to have a more significant impact on muscle fitness. Improvement in muscle fitness is affected by resistance exercise [52]. According to the recommendations of the ACSM, to avoid overtraining and to achieve the maximum benefits of resistance training, resistance training for the same muscle group should be separated by 48 h [17], which limits the frequency of physical activity. However, for muscle strength training, it has been shown that, although low-frequency training (once a week) may be enough to improve muscle strength after a few weeks, higher-frequency training may be more conducive to gaining muscle strength [53]. Most of the included studies used two to three sessions of physical activity per week, and no studies performed more than three or less than two sessions of exercise per week. Therefore, there is a need to investigate the effect of training frequency further.

#### 4.2.2. Training Intensity

Physical activity with high intensity enhanced muscle fitness well, which was consistent with current concepts [54,55,56]. For example, a systematic review of school-aged children and adolescents showed that high-intensity physical activity can better achieve health benefits in terms of strengthening muscle [54]. Moreover, an RCT of overweight and obese children illustrated that 12 weeks of high-intensity interval training was effective in improving lower limb muscle strength [57]. Similar results have been found in adolescents [58], adults [56] and older individuals [59]. In addition, it turned out that low-to-moderate intensity physical activity was also effective in improving muscle strength, which was supported by previous studies. Research conducted by David and colleagues confirmed that low-intensity weight training combined with plyometrics is effective in improving the muscle strength of young football players [60]. Another study of velocity-based resistance training with moderate intensity has yielded similar results [61]. It is worth noting that different types of physical activity may be affected by intensity differently, but the number of included literatures were not sufficient to support the discussion of intensity after type classification. Future studies can go further in this direction if possible.

#### 4.2.3. Training Duration

Physical activity of a duration less than 60 min in a single session was more conducive to improving muscle power. The improvement of muscle explosive power mainly depends on the load of the muscles during exercise and the speed at which the exercise is completed [62]. Consequently, for the same training volume, short-term exercise is more inclined to improve muscle explosive power [63]. In contrast, improvement of muscle endurance requires more repetitions, which require a longer training time [64]. However, our results showed that physical activity of a long duration per session did not improve muscle endurance. This may be because the studies included in this analysis had a variety of physical activity forms and could not guarantee completion of repeated stimulation targeting the same muscle group. Therefore, it was difficult to reflect the positive effects of physical activity on the improvement of muscle endurance.

#### 4.2.4. Training Type

In general, both resistance and non-resistance exercises play an important role in improving muscle strength and explosive power. This result supports the view that muscle fitness can be improved through various types of physical activity [65]. In terms of improving muscle endurance, resistance training may be more beneficial than non-resistance training, which is similar to the research of prior [66]. Furthermore, since there are few studies on the effects of muscle endurance, it should be interpreted with caution.

#### 4.2.5. Training Volume

For resistance exercise, physical activity with more than three sets and fewer than 10 repetitions per set was more beneficial to improve muscle fitness; a rest time of shorter than 120 s between helped to improve muscle endurance, while a rest time of more than 120 s helped to improve explosive power. This is consistent with the specific requirements of training for muscle strength, endurance, and explosive power. The improvement in muscle fitness is the result of continuous stimulation of muscle contraction at an appropriate load [67]. Many physical activity programs met the requirements for repeated stimulation. At the same time, taking the percentage of 1 RM as an indicator of intensity, programs with low repetitions often represent higher exercise intensity [68]. Both are beneficial for improving muscle fitness. In addition, the improvement of muscle endurance usually requires the next training to be performed before the effect of the previous training has completely disappeared, to achieve the effect of excessive recovery [69]. As the training of muscle explosive power requires instantaneous and marked contraction of muscles, it may cause a greater load on muscle fibers [70]. The next exercise needs to be performed after the effects of the previous exercise have completely disappeared to avoid injury [71]. Therefore, the rest time between groups should be reasonably chosen according to the different training objectives.

For non-resistance physical activity, training volume less than 500 METs-min/week improved explosive power, while more than 500 METs-min/week improved muscle strength. This may be because the proportion of high-intensity exercise in a general physical activity program is relatively small [72], so that the energy expenditure due to high-intensity exercise is less. Simultaneously, there may be less repetitive stimulation of the same muscle group in the general exercise program, and thus the improvement of muscle fitness is mainly reflected in explosive power. Physical activity with a high training volume usually involves 1 or more points of high frequency, intensity, and duration. Previous studies have shown that these three elements play a role in improving muscle strength. Farinatti et al. [73] conducted a study on the effect of different frequencies of training on female muscle strength and found that a higher weekly frequency increased muscle strength to a greater extent than a lower frequency of training. Furthermore, Jaswinder et al. [74] used accelerometers to monitor the daily exercise load of healthy women and found that high-intensity physical activity had a beneficial effect on muscle strength and bone density.

### 4.3. Theory Support

Several existing theories and studies could explain the relationship between physical activity factors and muscle fitness. The reason why high intensity physical activity was more effective seems to be explained by the supercompensation theory. On the one hand, muscle glycogen supercompensation increased after high intensity exercise [75]. On the other hand, high intensity training could induce HSP70, which may play an important role in muscle strength in response to exercise, and may increase the repetitions at 50% of 1-RM after high intensity training [76]. From the perspective of the theory of planned behavior [77], low frequency and short duration exercise was more receptive for children and adolescents and promote their behavioral intentions, which is one of determinants of participating in physical activity [78]. Although these theories supported the results of this study, more intensive mechanism research were needed to explore the relationship between physical activity factors and muscle fitness.

### 4.4. Limitations

This study had some limitations. First, the methodological quality of the research included in this study was not high (only one reached a Jadad score of ≥ 3). In addition, some studies did not report the data necessary for calculating the physical activity volume. Therefore, high methodological quality studies presenting the necessary data are needed to deepen our knowledge of physical activity in children and adolescents and to estimate the effects of physical activity on muscle fitness. Second, a general problem is the lack of reporting of physical activity intensity. Although the intensity of physical activity was divided into low to moderate and high according to the 2011 Compendium of Physical Activities, this may still affect the results of the analysis of the intensity and volume of physical activity to some extent. Therefore, we believe that actual exercise intensity of physical activity, by specifying or monitoring intensity, should be reported in future research. Third, this study lacked the analysis of fatigue and velocity loss in the set as the decisive characteristics of training load. As is acknowledged that fatigue is an important factor affecting training, and the influence of velocity inset on performance improvement also cannot be ignored. However, the literature included in this study rarely mentioned these two points, so we could not conduct further analysis. Further limitation is that this analysis is based on studies with different combinations of physical activity elements, and cannot provide insights about the interaction between each element. Thus, it is still unclear whether the performance gain would be maximal if the suggested range of all physical activity element in this study were implemented. Future research is supposed to determine the interaction between various elements through more effective analysis methods.

## 5. Conclusions

Physical activity plays an important role in improving the fitness of children and adolescents. By analyzing all physical activity elements concurrently, this study was able to suggest specific conditions that may benefit specific aspects of muscle fitness in children and adolescents. In order to help children and adolescents obtain better muscle fitness, there are several practical suggestions worth considering. First, promoting education reform to reduce the burden on students, decrease excessive school time occupation, and provide opportunities for increased physical activity. Second, implement the policy integrating sports and education as well as promote collaboration between home, school and society. For example, conducting scientific and high-quality physical education classes in schools, providing families with appropriate physical activity guidelines and increasing the construction of sports facilities to support the community with interesting and diversified physical activities.

Due to the low quality of the included literature, future study should be more rigorous in the research design. Meanwhile, many researches lack the description of fatigue degree and velocity loss in a set, so it may be a good direction to analyze the determining characteristics of training load through speed or fatigue degree control in training. In addition, further analysis of the linear relationship between physical activity elements and the muscle fitness would provide more theoretical support for the improvement of muscle fitness in children and adolescents.

## Figures and Tables

**Table 1:** Included studies examining the effects of physical activity in children and adolescence.

Author, Age	N Con	N Exp	Age	Subject	Sex	Progress	Frequency	Intensity	Time	Form	Sets	Reps	Rests	Volume	Jaded Score
**Resistance training**															
**Faigenbaum, 2001** [29]	12	IG1: 15 IG2: 16	8.1 ± 1.6	children	both	8	2	IG1: high IG2: moderate	NA	resistance training	NA	NA	NA	NA	1
**Harries, 2018** [30]	15	IG1: 8 IG2: 8	IG1: 16.8 ± 1.0 IG2: 17.0 ± 1.1 CG: 15.5 ± 1.0	rugby union players	male	12	2	moderate	60	resistance training	4~6	IG1: 3-10 IG2:3-5	NA	NA	2
**Moraes, 2013** [31]	10	IG1: 14 IG2: 14	IG1: 15.5 ± 0.9 IG2: 15.4 ± 1.1 CG: 15.6 ± 0.9	adolescence	male	12	3	67–75% 1RM	NA	resistance training	3	10~12	60–120	NA	2
**Nichols, 2001** [15]	11	5	14–17	students	female	60	3	75–77% 1RM	NA	resistance training	2~3	9~10	NA	NA	2
**Rey, 2017** [32]	16	IG1:16 IG2:15	IG1:17.4 ± 0.8 IG2:17.3 ± 0.8 CG:17.3 ± 0.9	soccer players	NA	10	2–3	NA	NA	eccentric hamstring training, Nordic curl, Russian belt	3	8~10	NA	NA	2
**Santos, 2008** [33]	10	15	CG:14.2 ± 0.4 IG:14.7 ± 0.5	basketball player	male	10	2	10RM	NA	resistance or plyometric training program	2~4	5~10	120–180	NA	2
**Santos, 2011** [34]	10	14	CG: 14.5 ± 0.4 IG: 15.0 ± 0.5	basketball players	male	10	2	NA	NA	in-season training program	4	6~10	180~240	NA	2
**Santos, 2012** [35]	10	15	CG: 14.2 ± 0.4 IG: 14.5 ± 0.6	basketball players	male	10	2	10RM	NA	resistance training	3	10	120–180	NA	2
**Szymanski, 2004** [36]	23	20	15.3 ± 1.1	baseball players	male	12	3	10 RM	NA	resistance training	2~3	10	90	NA	2
**Szymanski, 2007** [37]	24	25	14–18y	baseball players	male	12	3	45–85%1RM	NA	medicine ball exercises	2	6~10	90	NA	2
**Winwood, 2019** [38]	25	14	14.3 ± 0.5	adolescents	male	7	2	NA	60	resistance Training	2~3	5/10~15	120	NA	2
**Non-resistance training**															
**Alves, 2016** [39]	44	IG1: 45 IG2: 39	10.91 ± 0.51	children	both	8	2	20 m shuttle run: 75%VO_2max_	NA	IG1: strength training before run IG2: run before strength training	NA	NA	NA	NA	2
**Faigenbaum, 2015** [40]	21	20	CG:9.6 ± 0.3 IG:9.5 ± 0.3	student	both	8	2	NA	15	FIT program	NA	NA	NA	240	2
**Ferrete, 2014** [41]	13	11	IG:9.32 ± 0.25 CG:8.26 ± 0.33	soccer players	NA	26	2	high	30	strength and high-intensity training	NA	NA	NA	480	2
**Granacher, 2011** [42]	15	15	CG:6.6 ± 0.5 IG:6.7 ± 0.5	student	both	4	3	progressively increased	60	Balance exercise	NA	NA	NA	774	1
**Richards, 2014** [43]	1185	IG1:70 IG2:79	11–14y	students	both	11	1	NA	40	Football game	NA	NA	NA	320	3
**Lubans, 2010** [44]	30	IG1: 22 IG2: 15	CG:14.5 ± 0.6 IG1: 14.9 ± 0.6 IG2: 15.1 ± 0.7	student	both	8	2	NA	45	Elastic tubing and free weights exercises	20	8–10	60–90	540	3
**Mayorga-VIGa, 2016** [45]	57	54	12–14	student	both	17	2	Moderate to high	50	Mixed training program	NA	NA	NA	580	2
**Rodríguez-Rosell, 2016** [46]	15	15	IG: 12.7 ± 0.5 CG:12.8 ± 0.5	soccer players	NA	6	2	45–58%1RM	35	Mixed training program	NA	NA	NA	350	2
**St Laurent, 2018** [16]	11	17	9.3 ± 1.5	children	both	6	2	NA	60	suspension-training movement program	NA	NA	NA	960	2
**Yohei, 2013** [47]	58	36	13.7 ± 0.6	Exercise habits	boy	8	5	body mass-based	3	body mass-based squat movement	NA	NA	NA	75	2

**Table 2:** Jadad scores of the reviewed studies.

	Radom			Blinding			Lost/Exit		Total
	Without/Unclear/False	Mentioned but No Specific Method	“Radom” and Describe the Correct Method	Without/False	Mentioned but No Specific Method	„Double blind” and Describe the Correct Method	Not Mentioned	Detailed Description of Cases and the Reasons	
**Faigenbaum, 2001**	+			+				+	1
**Harries, 2018**		+		+				+	2
**Moraes, 2013**		+		+				+	2
**Nichols, 2001**		+		+				+	2
**Rey, 2017**		+		+				+	2
**Santos, 2008**		+		+				+	2
**Santos, 2011**		+		+				+	2
**Santos, 2012**		+		+				+	2
**Szymanski, 2004**		+		+				+	2
**Szymanski, 2007**		+		+				+	2
**Winwood, 2019**		+		+				+	2
**Alves, 2016**		+		+				+	2
**Faigenbaum, 2015**		+		+				+	2
**Ferrete, 2014**		+		+				+	2
**Granacher, 2011**	+			+				+	1
**Justin, 2014**		+			+			+	3
**Lubans, 2010**			+	+				+	3
**Mayorga-Vega, 2016**		+		+				+	2
**Rodríguez-Rosell, 2016**		+		+				+	2
**St Laurent, 2018**		+		+				+	2
**Yohei, 2013**		+		+				+	2

## Data Availability

The data presented in this study were obtained from the included studies and are openly available.

## Supplementary Materials

The following are available online at https://www.mdpi.com/article/10.3390/ijerph18189640/s1, Table S1: Coding for energy expenditure of non-resistance training; Figure S1: Funnel plot of publication bias.

## Registration and Protocol

The register name: The effect of physical activity elements on skeletal muscle fitness in children and adolescents: a meta-analysis of randomized controlled trials; The register number: CRD42020206963; Protocol access URL: PROSPERO International prospective register of systematic reviews. Available online: https://www.crd.york.ac.uk/PROSPERO (accessed on 2 September 2021); Protocol information amendments: title change from “The effect of physical activity elements on skeletal muscle fitness in children and adolescents: a meta-analysis of randomized controlled trials” to “The effect of intensity, frequency, duration and volume of physical activity in children and adolescents on skeletal muscle fitness: a systematic review and meta-analysis of randomized controlled trials”.

## References

[1] Wind A.E., Takken T., Helders P.J., Engelbert R.H. (2010). Is grip strength a predictor for total muscle strength in healthy children, adolescents, and young adults?. Eur. J. Pediatr..

[2] Volaklis K.A., Halle M., Meisinger C. (2015). Muscular strength as a strong predictor of mortality: A narrative review. Eur. J. Intern. Med..

[3] Castro-Piñero J., Perez-Bey A., Cuenca-Garcia M., Cabanas-Sanchez V., Gómez-Martínez S., Veiga O.L., Marcos A., Ruiz J.R., Marcos A., Gomez-Martinez S. (2019). Muscle fitness cut points for early assessment of cardiovascular risk in children and adolescents. J. Pediatr..

[4] Smith J.J., Eather N., Morgan P.J., Plotnikoff R.C., Faigenbaum A.D., Lubans D.R. (2014). The health benefits of muscular fitness for children and adolescents: A systematic review and meta-analysis. Sports Med..

[5] Ortega F.B., Ruiz J.R., Castillo M.J., Sjöström M. (2008). Physical fitness in childhood and adolescence: A powerful marker of health. Int. J. Obes..

[6] Mota J., Vale S., Martins C., Gaya A., Moreira C., Santos R., Ribeiro J.C. (2010). Influence of muscle fitness test performance on metabolic risk factors among adolescent girls. Diabetol. Metab. Syndr..

[7] Steene-Johannessen J., Kolle E., Andersen L.B., Anderssen S.A. (2013). Adiposity, Aerobic Fitness, Muscle Fitness, and Markers of Inflammation in Children. Med. Sci. Sports Exerc..

[8] Ortega F.B., Silventoinen K., Tynelius P., Rasmussen F. (2012). Muscular strength in male adolescents and premature death: Cohort study of one million participants. BMJ.

[9] Dong Y., Lau P.W., Dong B., Zou Z., Yang Y., Wen B., Ma Y., Hu P., Song Y., Ma J. (2019). Trends in physical fitness, growth, and nutritional status of Chinese children and adolescents: A retrospective analysis of 1.5 million students from six successive national surveys between 1985 and 2014. Lancet Child Adolesc..

[10] Tomkinson G.R. (2007). Global changes in anaerobic fitness test performance of children and adolescents (1958–2003). Scand. J. Med. Sci. Sports.

[11] Malina R.M., Katzmarzyk P.T. (2006). Physical activity and fitness in an international growth standard for preadolescent and adolescent children. Food Nutr. Bull..

[12] Tremblay M.S., Shields M., Laviolette M., Craig C.L., Janssen I., Gorber S.C. (2010). Fitness of Canadian children and youth: Results from the 2007-2009 Canadian Health Measures Survey. Health Rep..

[13] Moliner-Urdiales D., Ruiz J., Ortega F., Jiménez-Pavón D., Vicente-Rodriguez G., Rey-López J., Martínez-Gómez D., Casajús J., Mesana M., Marcos A. (2010). Secular trends in health-related physical fitness in Spanish adolescents: The AVENA and HELENA studies. J. Sci. Med. Sport.

[14] Seaborne R.A., Strauss J., Cocks M., Shepherd S., O’Brien T.D., Van Someren K.A., Bell P.G., Murgatroyd C., Morton J.P., Stewart C.E. (2018). Human skeletal muscle possesses an epigenetic memory of hypertrophy. Sci. Rep..

[15] Nichols D.L., Sanborn C.F., Love A.M. (2001). Resistance training and bone mineral density in adolescent females. J. Pediatr..

[16] St Laurent C.W., Masteller B., Sirard J. (2018). Effect of a Suspension-Trainer-Based Movement Program on Measures of Fitness and Functional Movement in Children: A Pilot Study. Pediatr. Exerc. Sci..

[17] The American College of Sports Medicine (ACSM) (2013). ACSM’s Guidelines for Exercise Testing and Prescription.

[18] Gebel A., Lesinski M., Behm D.G., Granacher U. (2018). Effects and dose–response relationship of balance training on balance performance in youth: A systematic review and meta-analysis. Sports Med..

[19] Lesinski M., Hortobágyi T., Muehlbauer T., Gollhofer A., Granacher U. (2015). Dose-response relationships of balance training in healthy young adults: A systematic review and meta-analysis. Sports Med..

[20] Lesinski M., Hortobágyi T., Muehlbauer T., Gollhofer A., Granacher U. (2015). Effects of balance training on balance performance in healthy older adults: A systematic review and meta-analysis. Sports Med..

[21] Nikolaidis P.T., Chtourou H., Torres-Luque G., Rosemann T., Knechtle B. (2019). The relationship of age and BMI with physical fitness in futsal players. Sports.

[22] Lesinski M., Prieske O., Granacher U. (2016). Effects and dose–response relationships of resistance training on physical performance in youth athletes: A systematic review and meta-analysis. Br. J. Sports Med..

[23] Powell K.E., Paluch A.E., Blair S.N. (2011). Physical activity for health: What kind? How much? How intense? On top of what?. Annu. Rev. Public Health..

[24] Moher D., Liberati A., Tetzlaff J., Altman D.G., Group P. (2009). Preferred reporting items for systematic reviews and meta-analyses: The PRISMA statement. PLoS Med..

[25] Ainsworth B.E., Haskell W.L., Herrmann S.D., Meckes N., Bassett D.R., Tudor-Locke C., Greer J.L., Vezina J., Whitt-Glover M.C., Leon A.S. (2011). 2011 Compendium of Physical Activities: A second update of codes and MET values. Med. Sci. Sports Exerc..

[26] Jadad A. (1996). Assessing the quality of reports of randomized clinical trials: Is blinding necessary?. Control. Clin. Trials..

[27] Cohen J. (2013). Statistical Power Analysis for the Behavioral Sciences.

[28] Higgins J.P., Thompson S.G. (2002). Quantifying heterogeneity in a meta-analysis. Stat. Med..

[29] Faigenbaum A.D., Loud R.L., O’Connell J., Glover S., O’Connell J., Westcott W.L. (2001). Effects of different resistance training protocols on upper-body strength and endurance development in children. J. Strength Cond. Res..

[30] Harries S.K., Lubans D.R., Buxton A., MacDougall T.H.J., Callister R. (2018). Effects of 12-Week Resistance Training on Sprint and Jump Performances in Competitive Adolescent Rugby Union Players. J. Strength Cond. Res..

[31] Moraes E., Fleck S.J., Ricardo Dias M., Simão R. (2013). Effects on strength, power, and flexibility in adolescents of nonperiodized vs. daily nonlinear periodized weight training. J. Strength Cond. Res..

[32] Rey E., Paz-Domínguez Á., Porcel-Almendral D., Paredes-Hernández V., Barcala-Furelos R., Abelairas-Gómez C. (2017). Effects of a 10-Week Nordic Hamstring Exercise and Russian Belt Training on Posterior Lower-Limb Muscle Strength in Elite Junior Soccer Players. J. Strength Cond. Res..

[33] Santos E.J., Janeira M.A. (2008). Effects of complex training on explosive strength in adolescent male basketball players. J. Strength Cond. Res..

[34] Santos E.J., Janeira M.A. (2011). The effects of plyometric training followed by detraining and reduced training periods on explosive strength in adolescent male basketball players. J. Strength Cond. Res..

[35] Santos E.J., Janeira M.A. (2012). The effects of resistance training on explosive strength indicators in adolescent basketball players. J. Strength Cond. Res..

[36] Szymanski D.J., Szymanski J.M., Molloy J.M., Pascoe D.D. (2004). Effect of 12 weeks of wrist and forearm training on high school baseball players. J. Strength Cond. Res..

[37] Szymanski D.J., Szymanski J.M., Bradford T.J., Schade R.L., Pascoe D.D. (2007). Effect of twelve weeks of medicine ball training on high school baseball players. J. Strength Cond. Res..

[38] Winwood P.W., Buckley J.J. (2019). Short-Term Effects of Resistance Training Modalities on Performance Measures in Male Adolescents. J. Strength Cond. Res..

[39] Alves A.R., Marta C.C., Neiva H.P., Izquierdo M., Marques M.C. (2016). Concurrent Training in Prepubescent Children: The Effects of 8 Weeks of Strength and Aerobic Training on Explosive Strength and VO2max. J. Strength Cond. Res..

[40] Faigenbaum A.D., Bush J.A., McLoone R.P., Kreckel M.C., Farrell A., Ratamess N.A., Kang J. (2015). Benefits of Strength and Skill-based Training During Primary School Physical Education. J. Strength Cond. Res..

[41] Ferrete C., Requena B., Suarez-Arrones L., de Villarreal E.S. (2014). Effect of strength and high-intensity training on jumping, sprinting, and intermittent endurance performance in prepubertal soccer players. J. Strength Cond. Res..

[42] Granacher U., Muehlbauer T., Maestrini L., Zahner L., Gollhofer A. (2011). Can balance training promote balance and strength in prepubertal children?. J. Strength Cond. Res..

[43] Richards J., Foster C., Townsend N., Bauman A. (2014). Physical fitness and mental health impact of a sport-for-development intervention in a post-conflict setting: Randomised controlled rial nested within an observational study of adolescents in Gulu, Uganda. BMC Public Health.

[44] Lubans D.R., Sheaman C., Callister R. (2010). Exercise adherence and intervention effects of two school-based resistance training programs for adolescents. Prev. Med..

[45] Mayorga-Vega D., Montoro-Escano J., Merino-Marban R., Viciana J. (2016). Effects of a physical education-based programme on health-related physical fitness and its maintenance in high school students: A cluster-randomized controlled trial. Eur. Phys. Educ. Rev..

[46] Rodríguez-Rosell D., Franco-Márquez F., Pareja-Blanco F., Mora-Custodio R., Yáñez-García J.M., González-Suárez J.M., González-Badillo J.J. (2016). Effects of 6 Weeks Resistance Training Combined With Plyometric and Speed Exercises on Physical Performance of Pre-Peak-Height-Velocity Soccer Players. Int. J. Sports Physiol. Perform..

[47] Yohei T., Yuko F., Eiji F., Hisashi M., Takaya Y., Masayoshi Y., Hiroaki K. (2013). Effects of Body Mass-Based Squat Training in Adolescent Boys. J. Sports Sci. Med..

[48] Fang H., Quan M., Zhou T., Sun S., Zhang J., Zhang H., Cao Z., Zhao G., Wang R., Chen P. (2017). Relationship between physical activity and physical fitness in preschool children: A cross-sectional study. BioMed Res..

[49] Feeney D., Stanhope S.J., Kaminski T.W., Machi A., Jaric S. (2016). Loaded vertical jumping: Force–velocity relationship, work, and power. J. Appl. Biomech..

[50] González-Badillo J.J., Rodríguez-Rosell D., Sánchez-Medina L., Ribas J., López-López C., Mora-Custodio R., Yañez-García J.M., Pareja-Blanco F. (2016). Short-term recovery following resistance exercise leading or not to failure. Int. J. Sports Med..

[51] Rodríguez-Rosell D., Mora-Custodio R., Franco-Márquez F., Yáñez-García J.M., González-Badillo J.J. (2017). Traditional vs. sport-specific vertical jump tests: Reliability, validity, and relationship with the legs strength and sprint performance in adult and teen soccer and basketball players. J. Strength Cond. Res..

[52] Granacher U., Lesinski M., Büsch D., Muehlbauer T., Prieske O., Puta C., Gollhofer A., Behm D.G. (2016). Effects of resistance training in youth athletes on muscular fitness and athletic performance: A conceptual model for long-term athlete development. Front. Physiol..

[53] Santos E.J., Janeira M.A. (2009). Effects of reduced training and detraining on upper and lower body explosive strength in adolescent male basketball players. J. Strength Cond. Res..

[54] Janssen I., LeBlanc A.G. (2010). Systematic review of the health benefits of physical activity and fitness in school-aged children and youth. Int. J. Behav. Nutr. Phys. Act..

[55] Buckley S., Knapp K., Lackie A., Lewry C., Horvey K., Benko C., Trinh J., Butcher S. (2015). Multimodal high-intensity interval training increases muscle function and metabolic performance in females. Appl. Physiol. Nutr. Metab..

[56] Cosgrove S.J., Crawford D.A., Heinrich K.M. (2019). Multiple fitness improvements found after 6-months of high intensity functional training. Sports.

[57] Cvetković N., Stojanović E., Stojiljković N., Nikolić D., Scanlan A., Milanović Z. (2018). Exercise training in overweight and obese children: Recreational football and high-intensity interval training provide similar benefits to physical fitness. Scand. J. Med. Sci. Sports.

[58] Lambrick D., Westrupp N., Kaufmann S., Stoner L., Faulkner J. (2016). The effectiveness of a high-intensity games intervention on improving indices of health in young children. J. Sports Sci..

[59] Kelly N.A., Ford M.P., Standaert D.G., Watts R.L., Bickel C.S., Moellering D.R., Tuggle S.C., Williams J.Y., Lieb L., Windham S.T. (2014). Novel, high-intensity exercise prescription improves muscle mass, mitochondrial function, and physical capacity in individuals with Parkinson’s disease. J. Appl. Physiol..

[60] González-Badillo J.J., Pareja-Blanco F., Rodríguez-Rosell D., Abad-Herencia J.L., del Ojo-López J.J., Sánchez-Medina L. (2015). Effects of velocity-based resistance training on young soccer players of different ages. J. Strength Cond. Res..

[61] Rodríguez-Rosell D., Franco-Márquez F., Mora-Custodio R., González-Badillo J.J. (2017). Effect of high-speed strength training on physical performance in young soccer players of different ages. J. Strength Cond. Res..

[62] Kawamori N., Haff G.G. (2004). The optimal training load for the development of muscular power. J. Strength Cond. Res..

[63] Tillin N.A., Folland J.P. (2014). Maximal and explosive strength training elicit distinct neuromuscular adaptations, specific to the training stimulus. J. Appl. Physiol..

[64] Desgorces F.D., Berthelot G., Dietrich G., Testa M.S. (2010). Local muscular endurance and prediction of 1 repetition maximum for bench in 4 athletic populations. J. Strength Cond. Res..

[65] Chen H., Chung Y., Chen Y., Ho S., Wu H. (2017). Effects of Different Types of Exercise on Body Composition, Muscle Strength, and IGF-1 in the Elderly with Sarcopenic Obesity. J. Am. Geriatr. Soc..

[66] Ivey F.M., Prior S.J., Hafer-Macko C.E., Katzel L.I., Macko R.F., Ryan A.S. (2017). Strength Training for Skeletal Muscle Endurance after Stroke. J. Stroke Cerebrovasc. Dis..

[67] Ratamess N.A., Alvar B.A., Evetoch T.E., Housh T.J., Ben Kibler W., Kraemer W.J., Triplett N.T. (2009). Progression models in resistance training for healthy adults. Med. Sci. Sports Exerc..

[68] Campos G.E., Luecke T.J., Wendeln H.K., Toma K., Hagerman F.C., Murray T.F., Ragg K.E., Ratamess N.A., Kraemer W.J., Staron R.S. (2002). Muscular adaptations in response to three different resistance-training regimens: Specificity of repetition maximum training zones. Eur. J. Appl. Physiol..

[69] Bellinger P. (2020). Functional Overreaching in Endurance Athletes: A Necessity or Cause for Concern?. Sports Med..

[70] Häkkinen K., Alen M., Kraemer W., Gorostiaga E., Izquierdo M., Rusko H., Mikkola J., Häkkinen A., Valkeinen H., Kaarakainen E. (2003). Neuromuscular adaptations during concurrent strength and endurance training versus strength training. Eur. J. Appl. Physiol..

[71] Hartmann H., Wirth K., Keiner M., Mickel C., Sander A., Szilvas E. (2015). Short-term periodization models: Effects on strength and speed-strength performance. Sports Med..

[72] Mullender-Wijnsma M.J., Hartman E., de Greeff J.W., Bosker R.J., Doolaard S., Visscher C. (2015). Improving academic performance of school-age children by physical activity in the classroom: 1-year program evaluation. J. Sch. Health..

[73] Farinatti P.T., Geraldes A.A., Bottaro M.F., Lima M.V.I., Albuquerque R.B., Fleck S.J. (2013). Effects of different resistance training frequencies on the muscle strength and functional performance of active women older than 60 years. J. Strength Cond. Res..

[74] Chahal J., Lee R., Luo J. (2014). Loading dose of physical activity is related to muscle strength and bone density in middle-aged women. Bone.

[75] Sonou T., Higuchi M., Terada S. (2008). An acute bout of high-intensity intermittent swimming induces glycogen supercompensation in rat skeletal muscle. Eur. J. Sport Sci..

[76] Liu Y., Lormes W., Wang L., Reissnecker S., Steinacker J.M. (2004). Different skeletal muscle HSP70 responses to high-intensity strength training and low-intensity endurance training. Eur. J. Appl. Physiol..

[77] Fishbein M., Ajzen I. (2011). Predicting and Changing Behavior: The Reasoned Action Approach.

[78] Fairchild Saidi G., Branscum P. (2020). Gender differences for theory-based determinants of muscle-strengthening physical activity in college-aged students: A moderation analysis. Transl. Behav. Med..

