# Supplementary material for: The Effect of Intensity, Frequency, Duration and Volume of Physical Activity in Children and Adolescents on Skeletal Muscle Fitness: A Systematic Review and Meta-Analysis of Randomized Controlled Trials
Source: Int J Environ Res Public Health. 2021 Sep 13;18(18):9640. doi: 10.3390/ijerph18189640 (PMC8468576; doi:10.3390/ijerph18189640)
Supplement: Supplementary file 1 [file ijerph-18-09640-s001.zip › ijerph-1315604-supplementary.pdf]

Table S1. Coding for energy expenditure of non-resistance training.

Author, year	Intensity	Physical activity	2011 Compendium of Physical Activities	
			Activity	MET
**Alves, 2016**	High	20 m shuttle run exercise and strength training	02040, circuit training, including kettlebells, some aerobic movement with minimal rest, general, vigorous intensity	8
**Faigenbaum, 2015**	High	FIT program	02040, circuit training, including kettlebells, some aerobic movement with minimal rest, general, vigorous intensity	8
**Ferrete, 2014**	High	Strength and high-intensity training program.	02020, calisthenics (e.g., push ups, sit ups, pull-ups, jumping jacks), vigorous effort	8
**Granacher, 2011**	Moderate	Balance exercises	02035, circuit training, moderate effort	4.3
**Justin, 2014**	High	Football game	15210, football, competitive	8
**Lubans, 2010**	High	Elastic tubing exercises and free weights exercises	02050, resistance training (weight lifting, free weight, nautilus or universal), power lifting or body building, vigorous effort (Taylor Code 210	6
**Mayorga-Vega, 2016**	Moderate	e.g. strength games, running games, circuit training, multi-jumps, or multi-throws	05180,walking/running, playing with child(ren), vigorous effort, only active periods	5.8
**Rodríguez-Rosell, 2016**	Moderate	strength training	02052, resistance (weight) training, squats , slow or explosive effort	5
**St Laurent, 2018**	High	suspension-training movement program	02020, calisthenics (e.g., push ups, sit ups, pull-ups, jumping jacks), vigorous effort	8
**Yohei, 2013**	Moderate	a body mass-based squat movement	02052, resistance (weight) training, squats , slow or explosive effort	5

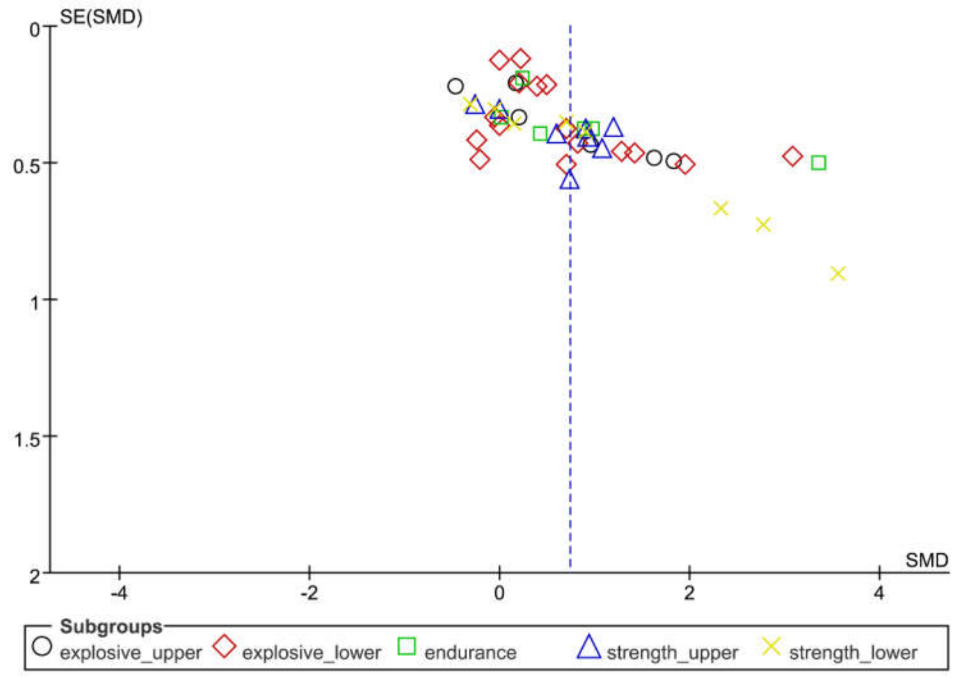

Figure S1. Meta-funnels**.**
